# BioASQ Synergy: a dialogue between question-answering systems and biomedical experts for promoting COVID-19 research

**DOI:** 10.1093/jamia/ocae232

**Published:** 2024-08-24

**Authors:** Anastasia Krithara, Anastasios Nentidis, Eirini Vandorou, Georgios Katsimpras, Yannis Almirantis, Magda Arnal, Adomas Bunevicius, Eulalia Farre-Maduell, Maya Kassiss, Vasileios Konstantakos, Sherri Matis-Mitchell, Dimitris Polychronopoulos, Jesus Rodriguez-Pascual, Eleftherios G Samaras, Martina Samiotaki, Despina Sanoudou, Aspasia Vozi, Georgios Paliouras

**Affiliations:** Institute of Informatics and Telecommunications, National Center for Scientific Research “Demokritos”, Athens 15341, Greece; Institute of Informatics and Telecommunications, National Center for Scientific Research “Demokritos”, Athens 15341, Greece; Department of Informatics, Aristotle University of Thessaloniki, Thessaloniki 54124, Greece; Institute of Informatics and Telecommunications, National Center for Scientific Research “Demokritos”, Athens 15341, Greece; Institute of Informatics and Telecommunications, National Center for Scientific Research “Demokritos”, Athens 15341, Greece; Institute of Biosciences and Applications, National Center for Scientific Research “Demokritos”, Athens 15341, Greece; Department of Biology “Charles Darwin”, Sapienza University of Rome, Rome 00185, Italy; Lithuanian University of Health Sciences, Kaunas 44307, Lithuania; Columbia University Vagelos College of Physicians and Surgeons, New York, NY 10033, United States; Barcelona Supercomputing Center, Barcelona 08034, Spain; NHS Improvement, London SE1 8UG, United Kingdom; Institute of Informatics and Telecommunications, National Center for Scientific Research “Demokritos”, Athens 15341, Greece; AstraZeneca Pharmaceuticals, Wilmington, DE 19803, United States; Ochre Bio Ltd, Oxford OX4 4GB, United Kingdom; Hospital Vithas Madrid la Milagrosa, Madrid 28010, Spain; St George’s University of London, London SW17 0RE, United Kingdom; Institute for Bioinnovation, BSRC “Alexander Fleming”, Vari 16672, Greece; Clinical Genomics and Pharmacogenomics Unit, 4th Department of Internal Medicine, Attikon Hospital, Medical School, National and Kapodistrian University of Athens, Athens 12462, Greece; Molecular Biology Division, Biomedical Research Foundation of the Academy of Athens, Athens 11527, Greece; Department of Informatics and Telecommunications, National and Kapodistrian University of Athens, Athens 16122, Greece; Institute of Informatics and Telecommunications, National Center for Scientific Research “Demokritos”, Athens 15341, Greece

**Keywords:** biomedical information, question answering, biomedical experts

## Abstract

**Objective:**

This article presents the novel BioASQ Synergy research process which aims to facilitate the interaction between biomedical experts and automated question-answering systems.

**Materials and Methods:**

The proposed research allows systems to provide answers to emerging questions, which in turn are assessed by experts. The assessment of the experts is fed back to the systems, together with new questions. With this iteration, we aim to facilitate the incremental understanding of a developing problem and contribute to solution discovery.

**Results:**

The results suggest that the proposed approach can assist researchers to navigate available resources. The experts seem to be very satisfied with the quality of the ideal answers provided by the systems, suggesting that such systems are already useful in answering open research questions.

**Discussion:**

BioASQ Synergy aspires to provide a tool that gives the experts easy and personalized access to the latest findings in a fast-growing corpus of material.

**Conclusion:**

In this article, we envisioned BioASQ Synergy as a continuous dialogue between experts and systems to issue open questions. We ran an initial proof-of-concept of the approach, in order to evaluate its usefulness, both from the side of the experts, as well as from the side of the participating systems.

## Introduction

Biomedical professionals have an extremely demanding schedule, with very limited time to follow scientific developments. This becomes a major challenge that can turn into a handicap, especially when taking into consideration the dizzying pace of these developments. As an example, the online biomedical bibliographic database PUBMED alone, currently comprises approximately 32 million references and has been growing at a rate often exceeding 20 000 articles per week (https://www.nlm.nih.gov/bsd/licensee/baselinestats.html).

Obtaining accurate and concise answers from this wealth of highly specialized information is a challenging task for traditional search engines. Instead of answers, such engines, return lists of potential relevant resources, mostly documents, that the experts have to evaluate. Automatically retrieving and extracting the required information is further complicated by nonstandard terminology and the ambiguity of the technical terms involved.

BioASQ has set up a challenge on biomedical semantic indexing and question answering (QA). Participating systems are required to semantically index content from large-scale biomedical sources (eg, MEDLINE; https://www.nlm.nih.gov/medline/medline\_overview.html) and to assemble data from multiple heterogeneous sources (eg, scientific articles, ontologies, databases) to compose informative answers to biomedical natural language questions. BIOASQ is driven by the crucial information management need of biomedical researchers: to synthesize and filter information from multiple, extremely large, and fast-growing sources.

Hence, BioASQ seeks to automate the information-seeking process that biomedical experts already use, that is, they need to express their information needs as natural language questions and receive answers, along with pointers to information sources supporting the answers. They should not have to manually specify related concepts (eg, corresponding to topics or synonyms of the terms used in their questions), which the systems may use internally. They should also not have to read entire documents when looking for a quick answer. Finally, by facilitating question-based queries, users no longer need to use specific query language and syntax.

The BioASQ challenge has been running since 2012 and has led to the formation of a very active community in biomedical QA. The challenge is designed to evaluate the performance of the participating systems and for this, it uses questions with widely recognized answers. BioASQ Synergy is an effort to go beyond this setup. Building upon the extended and highly active BioASQ ecosystem, it brings together biomedical experts and QA systems in a dialogue that aims to answer open research questions. The initial area of focus for this dialogue has been the COVID-19 pandemic.

In this article, we introduce the novel BioASQ Synergy research process and explain how the BioASQ infrastructure was used to facilitate the interaction between experts and systems. In particular, we present the new Synergy task of BioASQ, which allows biomedical experts to pose unanswered questions for developing problems. Participating systems attempt to provide answers to these questions, which in turn are assessed by the experts. The assessment of the experts is fed back to the systems, together with possibly new questions. Through this process, we aim to facilitate the incremental understanding of a developing problem and contribute to the discovery of new solutions.

The following section briefly introduces the BioASQ challenge. In the section “Introducing BioASQ Synergy”, the BioASQ Synergy process is explained, including the expert team, the infrastructure, and the datasets. The section “Evaluation” presents the evaluation approach, while the section “Results” presents the conclusions of this study.

## The BioASQ challenge

BioASQ is an international challenge on large-scale biomedical semantic indexing and QA. It has been running for 10 years and has created a very active ecosystem of Artificial Intelligence participant teams. These research teams make use of the BioASQ benchmarks and help push the state of the art in biomedical semantic indexing and QA. The impact of BioASQ in the field has been acknowledged by academic and corporate stakeholders, among which the US National Library of Medicine.

The QA task of BioASQ (Task B) takes place every year in 5 batches, released biweekly. For each batch, a trained team of biomedical experts (the BioASQ experts) provides about 100 new real-life research questions that the participating systems need to answer. In order to do so, the systems make use of the biomedical literature available in MEDLINE. Together with the questions, the BioASQ experts provide relevant MEDLINE documents and snippets, as well as the expected answers. The curation process is assisted by the BioASQ annotation tool.

Once the challenge is over, the experts assess the responses of the systems to their questions and they have the chance to enrich and improve the material that they have already provided. The assessment of the results is facilitated by the BioASQ assessment tool, which has a similar look-and-feel as the annotation tool. The result of the process is the annual QA benchmark of BioASQ. So far, the BioASQ QA benchmarks have accumulated 4721 questions, together with the associated material.

The challenge has significantly advanced the state-of-the-art over the years. In recent years, proposed approaches have leveraged state-of-the-art neural architectures such as BERT, PubMedBERT, BioBERT, and BART, adapting them to the biomedical domain and specifically to BioASQ tasks. Over the past year, several teams have explored methods based on large language models (LLMs), predominantly generative pre-trained transformer models, for the BioASQ tasks.^1–3^

**Figure 1.**
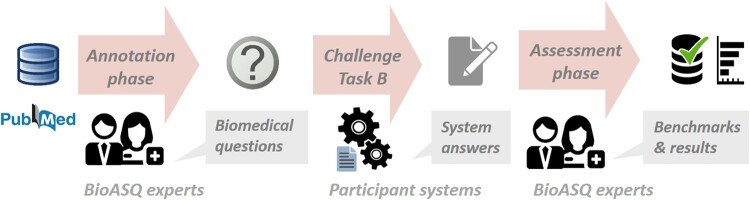
The workflow of task B, the BioASQ task on biomedical question answering.

The QA task of BioASQ is structured in a sequence of phases (Figure 1). First comes the annotation phase; then with a partial overlap runs the challenge; and only when this is finished starts the assessment phase. This leads to minimal interaction between the experts and the participating systems, which is acceptable due to the nature of the questions that are generated. Namely, in BioASQ task B we are looking for interesting research questions that have widely accepted answers.

## Introducing BioASQ Synergy

**Figure 2.**
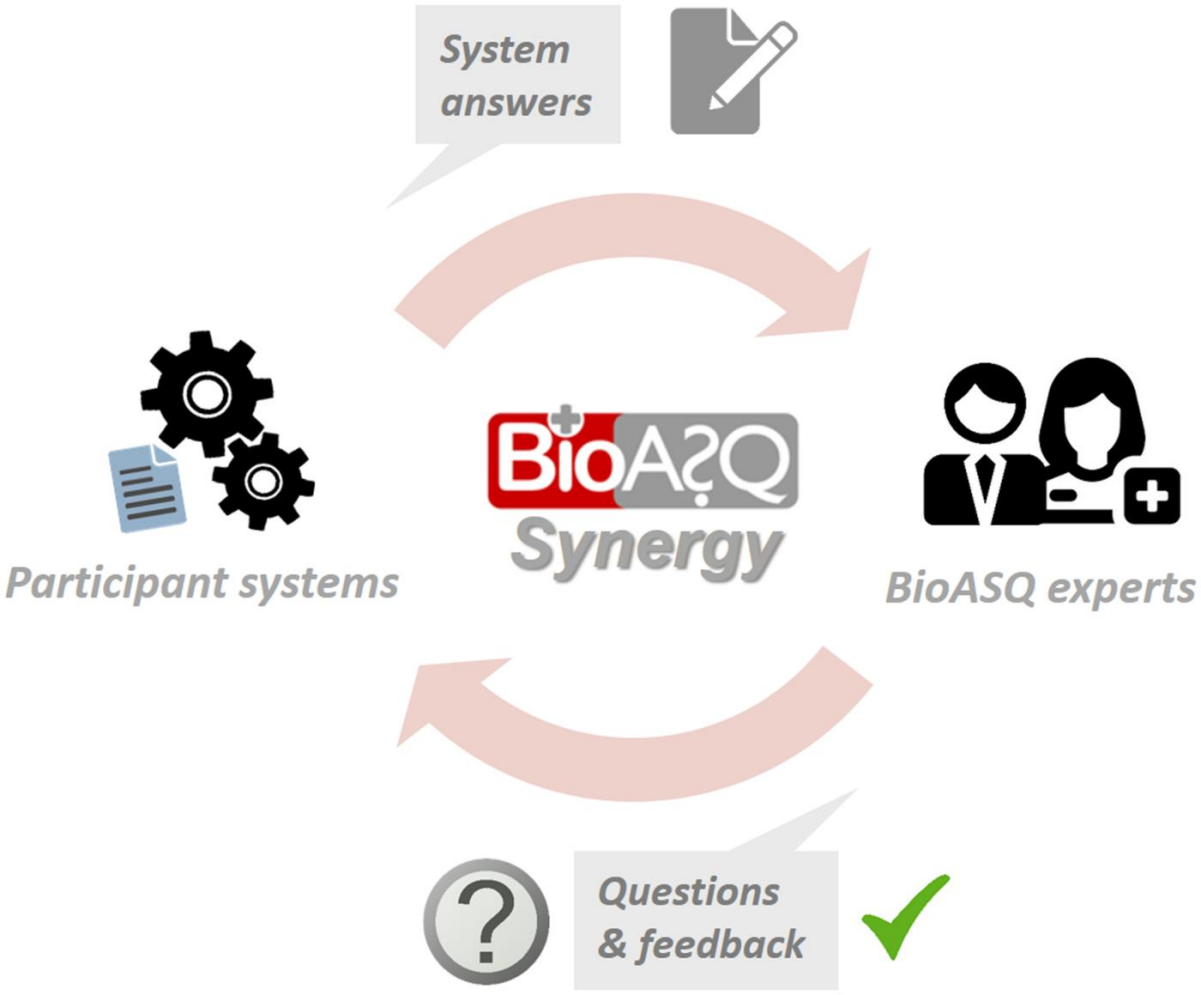
The iterative dialogue between the experts and the systems in the BioASQ Synergy task on question answering for COVID-19.

The above workflow is less suitable for fast-emerging biomedical research topics, such as COVID-19, where new issues appear every day and most of them remain open for some time. A more interactive approach is needed for such cases, facilitating a synergy between the biomedical experts and the automated QA systems. We envision such an approach as a continuous dialogue, where experts issue open questions, the systems respond to the questions, the experts assess the responses, their assessment is fed back to the systems, in order to help improve them, and the process continues. Figure 2 sketches this vision, in the form of a new BioASQ task.

The new task started in late 2020 and has run 3 times since then. Each version consisted of 4 biweekly rounds. In particular, the first version took place beginning December 2020, May 2021, and December 2022. In each round, a set of COVID-19 questions was given to the participating teams, who employed their systems to retrieve relevant documents and snippets and to identify answers to the questions. Both short answers, such as entity names, and paragraph-sized answers in English were accepted. All the teams were required to submit their responses in a limited time period, and only material from the current version of the CORD-19 dataset^4^ was considered. The experts assessed the submitted material (documents and snippets) and answers, and provided feedback to the systems, so that they could improve their responses. This process proceeded with new feedback and new predictions from the systems in an iterative way.

Each team was free to participate in any of the rounds and response types. For example, some teams could focus on developing comprehensive systems that provide both relevant material and answers, whereas other teams could choose to focus on approaches that identified relevant documents or on generating answers in natural language only. This was possible, as the same expert feedback was provided to all participants, based on the submissions received in the previous rounds. As a result, a team could participate in the generation of answers, without having to retrieve relevant documents and snippets. Instead, they could include the ones in the experts’ feedback.

The task was organized as part of the ninth and tenth editions of the BioASQ challenge and the approaches of the participating teams were presented in the BioASQ 2021^5^ and 2022^6^ workshop, in the context of CLEF 2021 and CLEF 2022, respectively. Next, we present the different aspects of the BioASQ Synergy approach, namely the expert team, the question generation and answer assessment process, the supporting tools, and the resulting dataset.

### BioASQ expert team

Due to the interactive workflow, biomedical experts are more involved in BioASQ Synergy than in BioASQ Task B. The team included experts from different regions and with a wide range of expertise, background, and experience. All experts had some interest in the developing topic of the COVID-19 pandemic. The aim was to have a variety of questions covering different aspects of the developing issue. In particular, the expert team included medical doctors and researchers from hospitals, research centers, and companies, working in diverse domains such as pharmacogenomics, immunology, bioinformatics, and clinical informatics.

The experts were free to pose any COVID-19-related question, whether it fell within their exact area of expertise or in a broader field of interest. In each round, the participating systems were retrieving material relevant to the question, as well as candidate answers, and the experts could choose which of them were satisfactory, if any. In total, the expert team consisted of 14 experts that contributed to the task by posing questions and assessing the system responses. In particular, 11 experts contributed to the first version of the task, 9 experts to the second version, and 7 in the third version. Five experts participated in all 3 versions. In addition, a coordinator with experience in the structure of BioASQ, inspected the questions and the assessed materials, in order to ensure that they adhere to the quality requirements and followed the guidelines provided by the organizers.

### Generating questions and assessing responses

The experts were asked to pose relevant questions about COVID-19, representing their actual information needs. Such questions could range from general to very specific. In addition, these questions could vary in difficulty and for some, the information available could be insufficient to provide a valid answer, since the problem was still developing. Furthermore, the experts could choose questions from a predefined stack provided for the TREC-COVID shared task.^7^ Each predefined question could be used by up to 2 experts.

When a question was first formulated, it was not yet clear if enough information was available. Hence, the systems were only asked to retrieve relevant documents and snippets at that stage. Next, after receiving the responses of the systems, experts marked as “golden” the documents and snippets that they considered relevant to their question. When the experts were confident that the retrieved material was sufficient for providing an answer to the questions, they marked the questions *ready to be answered*.

Following the assessment phase, the experts’ feedback was provided to the systems, in order to improve their performance and generate new responses for the same questions. Thus, in the next round the systems generated new documents and snippets for the questions while, for those questions marked as *ready to be answered*, the systems could also provide answers. The material generated by the participating systems, including any answers, was again assessed by the experts and their feedback was given to the systems.

During the assessment of any round, if an expert was confident that a question had been fully answered and the answer is not expected to change, they could choose to “*close*” a question. Closing a question meant that it would not appear in any following rounds. Nevertheless, a question could stay open for many rounds and versions of the task, because, answering a question related to an ongoing problem is not a deterministic task and the answers may change in between weeks or rounds. For example, the answers to “What are the available vaccines for COVID-19?,” changed radically in the course of the task, as new articles and indeed vaccines became available.

Given the dynamic nature of topics like COVID-19, some questions could branch out to new directions. Hence, if an expert believed that an existing question had a new interesting angle, they could choose to create a “*new version*” of it. Creating a new version of a question meant that a copy of the previous question was created. In the new version of the question, the type and body could change, there would be no answers associated with it, but it would inherit all golden documents and snippets from the original question. Hence the expert may rethink and re-ask a slightly modified version of the question, based on the answers and the overall retrieved content of the systems. For example, one could transition from “Are there any COVID-19 vaccines?” to “What are the available vaccines for COVID-19?”.

The questions and the assessment of their answers are highly dependent on the personal perception of the expert. The task is structured so that each expert generates their own questions based on their individual preferences. Additionally, because the questions are open-ended and dynamic, the standards for what constitutes a satisfactory answer can differ among experts. As a result, BioASQ Synergy does not aspire to provide an objective evaluation of the performance of the systems, but rather to be used as *a tool that gives the experts easy and personalized access to the latest findings in a fast-growing corpus of material.*

### The BioASQ Synergy tool

To accommodate the needs of BioASQ Synergy we developed a versatile, easy-to-use tool, serving both the experts and the BioASQ organizing team (http://at.bioasq.org/). The tool was based on the annotation and assessment tools, used in BioASQ Task B, extended to provide the required additional flexibility. In particular, the BioASQ Synergy tool provides the necessary functionality to create questions, as well as review the documents, snippets, and answers returned by the systems, and to annotate them as relevant or not. This information is communicated back to the systems, which try to take it into account and improve their answers in the next round.

**Figure 3.**
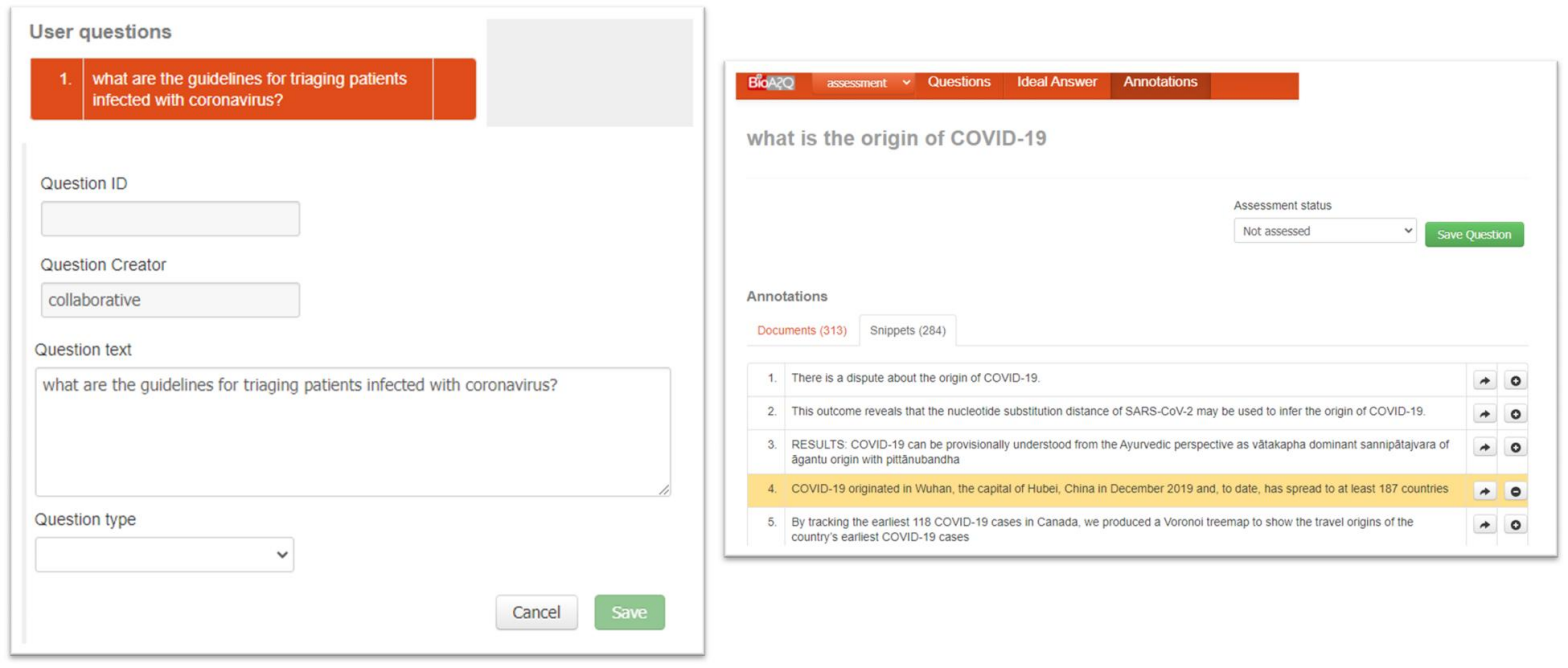
An example of a question creation (left). Assessing results for the question: “What is the origin of COVID-19?” (right).

Figure 3 (left) provides an example question. The expert formulates the question and select its type. Figure 3 (right) illustrates part of the assessment process of a question. The documents and snippets provided by the systems are presented. The experts give their feedback, by marking the relevant material (the yellow snippet in this example).

### Dataset description

The data generated during the first 3 versions of BioASQ Synergy for COVID-19 include questions, related material, and where available answers. Information on the evolution of the material collected during the task for each question is also included in the dataset.

Each question belongs to 1 of 4 types:

- Yes/no questions: These are questions that require “yes” or “no” answers. For example, “Have there been network-based drug repurposing approaches for SARS-CoV-2?” is a yes/no question.
- Factoid questions: These are questions that require a particular entity name (eg, of a disease, drug, or gene), a number, or a similar short expression as an answer. For example, “Which gene cluster has been identified as a genetic susceptibility locus in patients with Covid-19 with respiratory failure?” is a factoid question.
- List questions: These are questions that require a list of entity names (eg, a list of gene names), numbers, or similar short expressions as an answer. For example, “Which central cellular pathways does SARS-CoV-2 reshape?” is a list question.
- Summary questions: These are questions that do not belong in any of the above categories and can only be answered by producing a short text summarizing the most prominent relevant information. For example, “Describe the role of neuropilin-1 (NRP1) in COVID-19” is a summary question.

**Figure 4.**
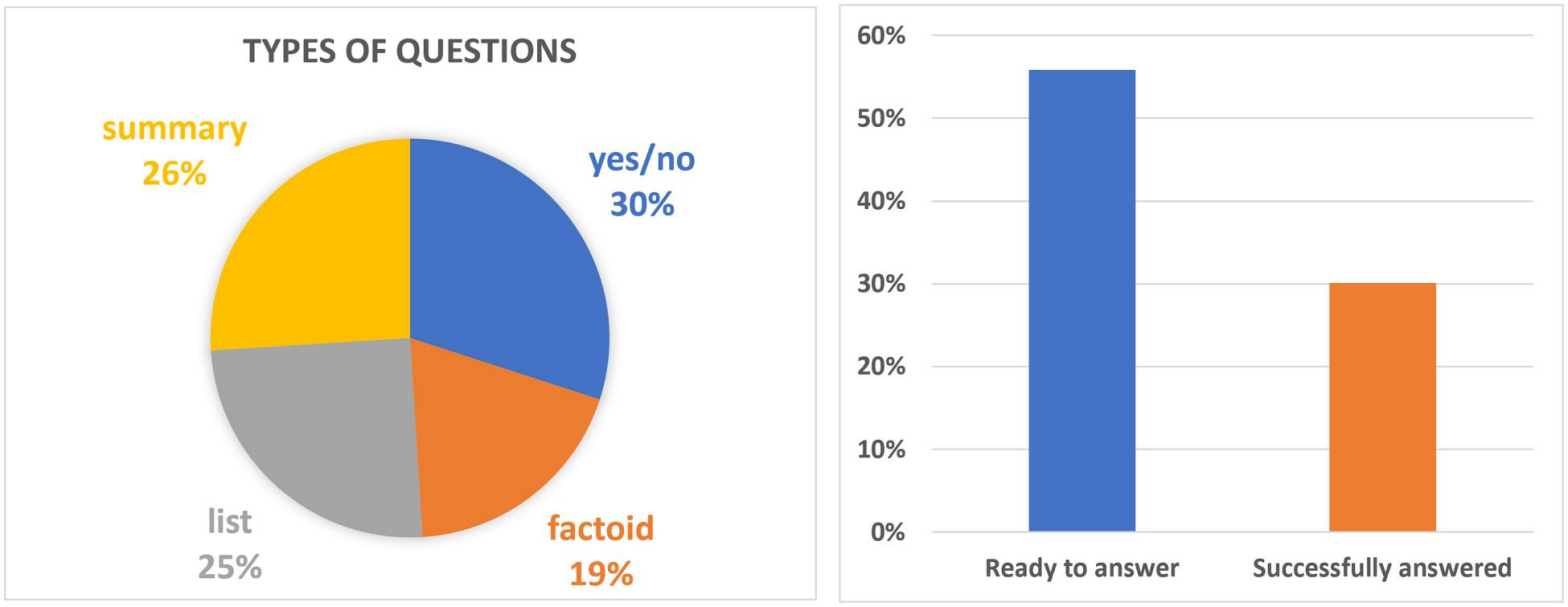
The figure on the left presents the percentages of the different types of questions in the dataset. The figure on the right presents the percentages of questions marked as *ready to answer* and the ones successfully answered after the final round of the third version.

Out of 256 unique questions in BioASQ Synergy so far, 30% of them were yes/no questions, 19% factoid, 25% list, and 26% summary (Figure 4). Fifty-six percent of them have been marked as ready to accept answers, that is, the experts judged that sufficient material has been accumulated, and 30% of them were answered successfully. For all question types, if the question is ready to answer, the dataset also contains a list of paragraph-sized texts ideally summarizing the most relevant information from articles and snippets, termed in BioASQ “*ideal answers*.”

The relevant articles to answer the questions have been retrieved from the CORD-19 dataset. The results of the traditional QA task in BioASQ (Task B) suggest that the title and abstract of MEDLINE documents are good resources for answering scientific biomedical questions, as they are always available and usually summarize the main contributions of each article. Hence, the task Synergy implementation has focused on scientific articles and relevant snippets extracted from their title and abstracts, achieving promising results based on the experts’ feedback. However, considering the full text and addressing relevant limitations of availability, formatting, and redistribution rights is an interesting future direction, compatible with the Synergy framework. A new version of the CORD-19 dataset was available at each round of the challenge, meaning that new information could be retrieved for existing questions. As mentioned in the section “Introducing BioASQ Synergy”, feedback from the experts was provided in each round. The feedback includes a collection of the participants’ responses for each question, with information on the relevance of the response to the answer to the question. In addition, the feedback included any exact/ideal answer submitted by the systems that were assessed as relevant by the experts (Table 1).

**Table 1.** Statistics of the datasets of the Synergy task.

Version	Round	Size	Yes/No	List	Factoid	Summary	Answer	Feedback
1	1	108	33	22	17	36	—	—
1	2	113	34	25	18	36	53	89%
1	3	113	34	25	18	36	80	86%
1	4	113	34	25	18	36	86	91%
2	1	95	31	22	18	24	6	17%
2	2	90	27	22	18	23	10	80%
2	3	66	17	14	18	17	25	100%
2	4	63	15	14	17	17	33	100%
3	1	72	21	20	13	18	13	36%
3	2	70	20	19	13	18	25	100%
3	3	70	20	19	13	18	41	100%
3	4	64	18	19	10	17	47	100%

“Answer” stands for questions marked as having enough relevant material from previous rounds to be answered. “Feedback” for questions that already have some expert feedback from previous rounds.

In each round, the system responses and expert feedback refer to the same questions, although some new questions or modified versions of previous questions could be added to the test sets. Overall, 14 questions were created as new versions of previous questions. For example, the initial question “*What is the rate of mother-to-child transmission of Covid-19?*” was posed in the first round of the second version of the task. Then, in the first round of the third version, the expert generated a new version of this question, namely “*Does Covid-19 in the mother affect the newborn?*,” considering the material and answers already gathered for the initial question at that point.

Beyond the first round, an average 91% of the questions received feedback in each round. On an average, 54.5 documents and 59.7 snippets were provided by the systems per round for each question. In each round, only the questions marked as *ready to answer* accepted exact/ideal answers by the systems. On average 19.8 questions were marked as *ready to answer* per round, and, at the end of the 3 versions, 196 questions were marked as *ready to answer* in total. The mean number of rounds required for each of these questions until it was marked as *ready to answer* was 2.2. For 97 of these questions, at least one satisfactory ideal answer was submitted by the systems, based on expert assessment. For the vast majority of them, about 97%, the first satisfactory ideal answer was retrieved directly in the same round when it was marked as *ready to answer*.

After receiving an answer, satisfactory or not, the question can remain active for potential new relevant material or updates. This was the case for most questions that received answers, as only about 12% of them were marked as “closed” after receiving their first satisfactory ideal answer. In a few cases, the systems did manage to retrieve additional answers in the following rounds. For the question “*What are the complications of COVID-19 Nasopharyngeal Swab Test?*,” for instance, only 6 complications were identified in the third and fourth rounds of the second version. In the new round of the third version, however, 2 additional complications were found, namely “asystole” and “syncope.”

Overall, each question remained active for about 4.1 rounds. The overlap between the first and the second version of Synergy was 13 questions, and the overlap between the second and the third version was 17 questions. The most extreme case was for the question “*What is the pathophysiological mechanism of hemostasis disorder in SARS-CoV-2 infection?*” which was posed in the first round of the first version, but no sufficient information was available for answering until the first round of the second version. Still, after receiving 6 alternative satisfactory ideal answers in that round, the question remained open until the end of the third version, as the question remained of interest for potential updates based on newer material. Indeed, 24 more satisfactory answers were gathered by the end of the task.

The details of the datasets used in each round of the 3 versions of Synergy that ran so far are provided in Table 1.

## Evaluation

### Methodology

The first version of BioASQ Synergy task started in December 2020 and ended in late January 2021, the second version took place between May 2021 and June 2021, and the third one started in December 2021 and ended in late January 2022. All datasets are publicly available through the BioASQ website (http://participants-area.bioasq.org/datasets/).

Similar to previous BioASQ challenges, the participating teams were allowed to upload multiple submissions in each round, in order to test different variants of their systems. Each team was permitted to submit responses from a maximum of 5 systems.

To properly assess the scoring performance of the systems, based on the feedback of the experts, we followed a procedure similar to the residual collection evaluation.^8^ This procedure relates to how we handled answers and related material (eg, documents) provided as feedback by the experts. Specifically, we did not evaluate the systems on the basis of supporting material, as we consider that the characterization of documents and snippets, as relevant or not to the question, cannot change over time. Hence, if a system provided a new judgment for such material, the response would be ignored. On the other hand, answers to questions could be submitted at any time, regardless of whether they are included in the feedback of previous rounds. This is because the correctness and completeness of an answer may change over time, as new knowledge becomes available.

The 3 versions of BioASQ Synergy consisted of 12 rounds overall, 4 rounds per version, and the evaluation was performed in each round separately. In total, 50 systems from 15 different teams participated. Of those teams, 4 participated in all 3 versions. An additional preparatory round was also organized, prior to the official versions, as a dry run for the participants to familiarize themselves with the procedure. There were 6 countries represented and all teams except from 2 affiliated to the industry, were affiliated to academic institutes. Additional information concerning the participation can be found in Refs.^5^^,^^6^

#### Evaluation metrics

Similar to BioASQ task B, the participating systems were evaluated on their ability to: (1) retrieve relevant material (articles and snippets), (2) identify “exact” answers, and (3) identify “ideal” answers, that is, narrative justification of the exact answer. Different evaluation metrics were used for each case.

Specifically, we used the mean average precision (MAP) measure and the mean F1-measure to evaluate the relevance of articles and snippets, respectively. For the sake of completeness, we also computed mean precision, mean recall, and mean F1-measure for each system (Full results are reported at http://participants-area.bioasq.org/results/). For document and snippet retrieval, the participating systems were expected to submit lists of at most 10 relevant articles (documents) and 10 relevant text snippets.

Regarding “exact” answers, we used different metrics depending on the type of the question under consideration. Therefore, for “yes/no” questions we calculated the accuracy and the macro F1-measure, but only the latter was considered for the final rankings. For “factoid” questions, each participating system had to return a list of up to 5 entity names (eg, up to 5 names of drugs), ordered by decreasing confidence. To evaluate this type of question, we measured the strict and lenient accuracy,^9^ as well as the mean reciprocal rank score, which was also used as the official ranking measure. For “list” questions, each participating system had to return a single list of entity names, jointly taken to constitute a single answer (eg, the most common symptoms of a disease). The returned list could not contain >100 entries of 100 characters maximum each. To evaluate these lists, we computed the MAP, recall, and F1-measure, the latter being used also for the rankings.

Lastly, for the “ideal” answers, the participating teams were expected to provide a single paragraph-sized text of maximum length of 200 words, ideally summarizing the most relevant information from articles and snippets. These summaries were evaluated in 2 ways: (1) automatically and (2) manually. For automated evaluation, we used ROUGE, a popular technique that measures the overlap between text summaries. Additionally, the BioASQ experts manually inspected the “ideal” answers of the participating systems, providing feedback along 4 different dimensions: precision, recall, repetition, and readability.

## Methods

The majority of the systems utilized state-of-the-art pretrained language representation models, including BERT,^10^ Roberta,^11^ and T5^12^ for their methods. An overview of systems and approaches employed in this task is provided in Table 2. Most of these methods are described in their respective published papers in the CLEF2021 (http://ceur-ws.org/Vol-2936/) and CLEF2022 (https://ceur-ws.org/Vol-3180/) proceedings, where the reader can find further information.

**Table 2.** Systems and their approaches for task Synergy.^6^

System	Approach
RYGH	BM25, BioBERT, PubMedBERT, T5, BERTMeSH, SciBERT
PSBST	BERT, SQuAD1.0, SpanBERT, XLNet, PubMedBERT, BioELECTRA, BioALBERT, BART
bio-answerfinder	Bio-ELECTRA++, BERT, weighted relaxed word mover’s distance (wRWMD), pyserini with MonoT5, SQuAD, GloVe
MQ	tf-idf, sBERT, DistilBERT
bioinfo	BM25, ElasticSearch, distant learning, DeepRank, universal weighting passage mechanism (UPWM), BERT

### Expert user evaluation

As mentioned above, BioASQ Synergy aspires to be more than a challenge. In particular, it aims to help biomedical experts in progressing their research. In order to assess the usefulness and potential of this novel approach, we collected feedback and opinions of the expert team, based on their experience in the 3 versions that have run so far. Specifically, we distributed a questionnaire, comprising questions on the usefulness of the tool, the overall experience, the performance of the systems, the improvement of the performance based on their feedback, and more. Overall, 18 responses were submitted (as some of the 14 experts, participated in more than one rounds of the task and evaluated the tool twice).

About half of the experts found BioASQ Synergy useful as a time-saving first step for investigating a question on a new domain, but the responses of the systems are not yet consistently good across different types of questions. Two-thirds of the experts found the performance of the systems satisfactory as a first-stage retrieval, often better than what they expected by an automated analysis of the literature. In particular, it seems that most experts found the performance on documents retrieval, snippet retrieval, and exact answer generation good or very good, whereas for ideal answers the performance appears to be less satisfactory. The latter seems to contradict the results presented in the previous section, but it is important to keep in mind that the experts assessed the responses of all participating systems, while the figures above present only the top-performing system. Additionally, the experts would like to have information on the quality and reliability of the sources of the information provided by the systems, which is not included in the Synergy framework yet.

In addition, BioASQ Synergy helped some experts to find interesting information or material that they were not aware of, particularly for questions in subareas that were beyond their exact expertise. This includes both specific information, such as which particular Asthma categories might be a risk factor for COVID-19, as well as more generic information, such as recent meta-analyses about relevant topics. About half of the experts noticed an improvement in the answers of the systems over time, which could be attributed to the feedback that the experts provided. On the other hand, most of the relevant material (documents and snippets) for the questions was often gathered from the initial round. In particular, only about 20% of the experts observed an important or very important improvement during the rounds, which can be related to new material becoming available, during the challenge.

All experts stated that they would be interested to incorporate the BioASQ Synergy platform into their daily research routine, rating their overall experience with an average score of 4 in a scale of 1-5. In particular, about 60% of the experts had a clearly positive overall opinion about BioASQ Synergy, while the average interest in participating in a future version and suggesting Synergy to a colleague were both about 4.4 in a scale of 1-5. In the Supplementary Appendix, figures which summarize most of the answers of the experts are presented.

## Results

**Figure 5.**
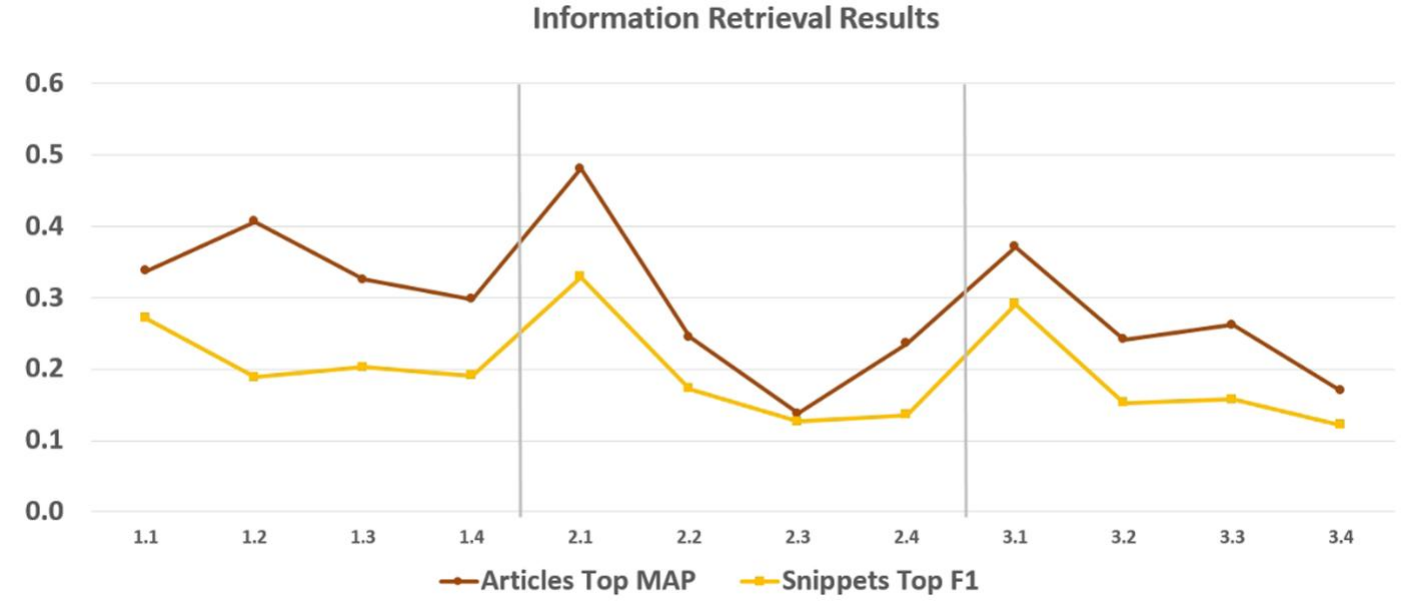
The best performances for the retrieval of documents and snippets.

Figure 5 shows the best performances achieved in document and snippet retrieval. Specifically, the top MAP scores for article retrieval varied between 0.15 and 0.5, while for snippets the top F1 scores varied between 0.1 and 0.35. Moving from version 1 to version 2, as well as from version 2 to version 3 of the task, we observe spikes in performance, followed by a steep fall. This is most probably related to the new material that became available in CORD-19, in the 3-month interval between the 3 versions.

**Figure 6.**
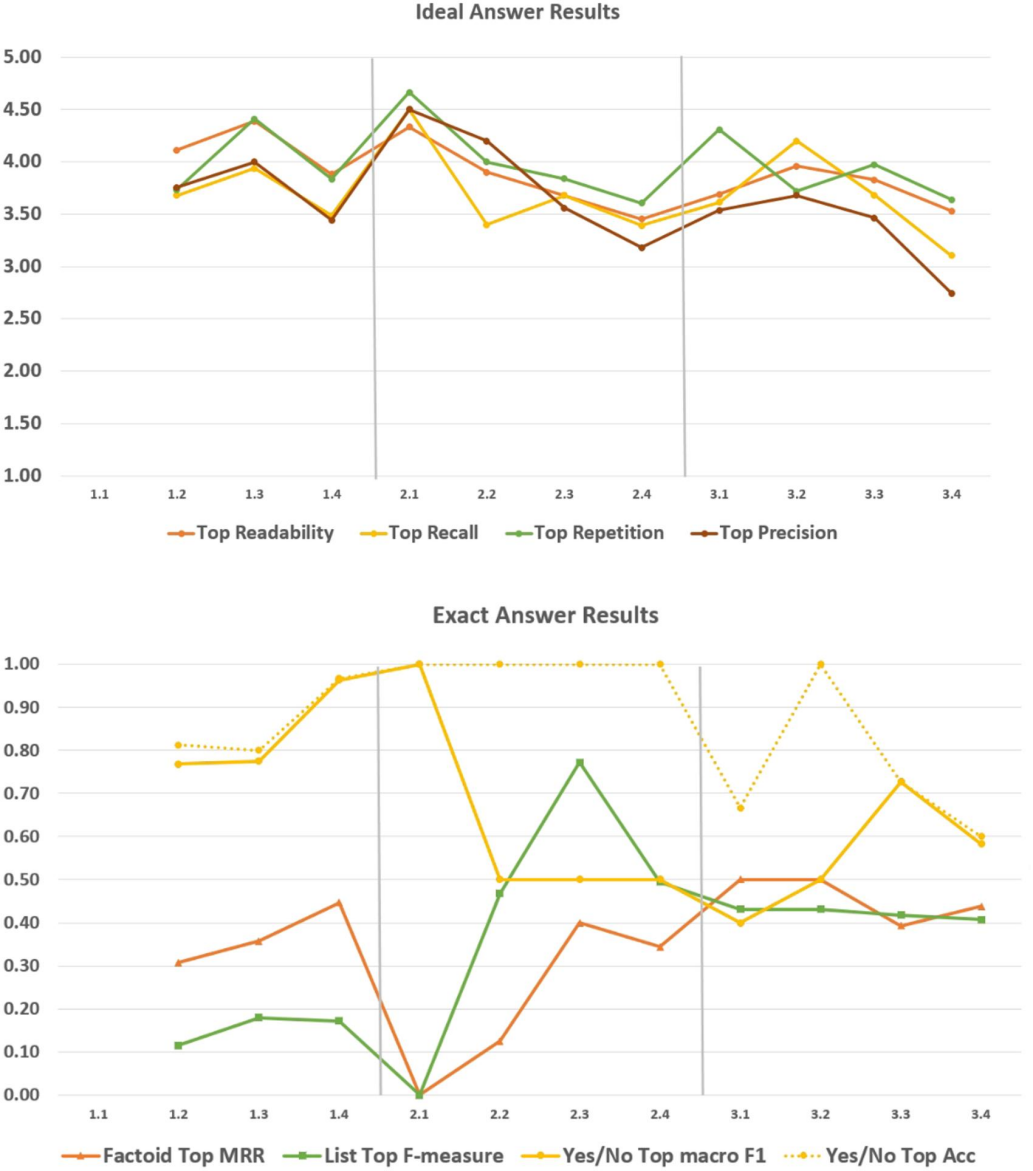
The best performances as reported for the “Ideal” and exact answers.

Figure 6 depicts the performance of the systems in providing “Ideal Answers.” In particular, we present the readability, recall, precision, and repetition scores for the highest-scoring systems. These scores represent the manual evaluation carried out by the BioASQ expert team and the results demonstrate that current systems are able to provide satisfactory short-summary answers for open biomedical questions.

Lastly, the results for providing the “exact” answers vary depending on the type of question. In particular, the performance of the participating systems for “factoid” questions ranged from 0.1 to 0.46 (Figure 6).

Overall, the results of the 3 versions of BioASQ Synergy are encouraging. There is clearly much room for improvement in both the retrieval of relevant documents and snippets, but also in generating exact answers. However, the experts seem to be very satisfied with the quality of the ideal answers provided by the systems, suggesting that such systems are already useful in answering open research questions. Hopefully, Synergy will set an important baseline for future experiments and motivate progress in similar open research topics.

## Conclusions

This article presents a novel approach to assist biomedical experts in answering open questions related to their research. We ran an initial proof-of-concept of the approach, in order to evaluate its usefulness, both from the side of the experts, as well as from the side of the participating systems. In particular, we introduced a new task in the BioASQ challenge, called BioASQ Synergy, which allows biomedical experts to pose unanswered questions for developing problems, such as COVID-19. Through this process, we aimed to facilitate the incremental understanding of a developing problem and contribute to the discovery of new solutions. Despite the fact that an active search for biomedical information requires an ulterior evaluation of the quality and importance of the sources, the results that we obtained suggest that the proposed approach can help researchers. Moreover, the challenge aims to lead to substantial progress in text generation tasks. This progress is particularly important in the era of LLMs, which have introduced new capabilities and techniques. New versions of the challenge are running. In the new versions of the Synergy task, the experts are free to ask questions about any emerging topic of their interest, and not solely focus on COVID-19.

## Supplementary Material

ocae232_Supplementary_Data

## Data Availability

The datasets generated during the current study are available at http://participants-area.bioasq.org/datasets/
